# Differential diagnosis of pigmented nail lesions^☆^

**DOI:** 10.1016/j.abd.2024.01.005

**Published:** 2024-08-06

**Authors:** Laura Bertanha, Leandro Fonseca Noriega, Nilton Gióia Di Chiacchio, Adriana Matter, Nilton Di Chiacchio

**Affiliations:** a Dermatology Service, Hospital do Servidor Público Municipal de São Paulo, São Paulo, SP, Brazil; b Dermatology Service, Faculdade de Ciências Médicas, Universidade Estadual de Campinas, Campinas, SP, Brazil; c Dermatology Service, Faculdade de Medicina do ABC, Santo André, SP, Brazil; d Dermatology Service, Hospital Santa Casa de Curitiba, Curitiba, PR, Brazil

**Keywords:** Nail diseases, Melanoma, Neoplasm, Dermoscopy, Hematoma, Onychomycosis

## Abstract

The diagnosis of pigmented nail lesions is a concern for both general practitioners and dermatologists, due to the possibility of indicating nail melanoma. The origin of the dark pigmentation can be either melanocytic or non-melanocytic (fungi, bacteria, or blood), and clinical evaluation alone may not be sufficient for differentiation, requiring additional exams. Onychoscopy provides valuable information prior to biopsy. The causes of nail pigmentation will be described to aid in the differential diagnosis.

## Introduction

Pigmented nail lesions can exhibit various colors and sizes, and their origin can be either melanocytic or non-melanocytic.^1^

In cases of non-melanocytic pigment lesions, such as bacterial or fungal infections and hematomas, clinical findings along with supplementary examinations like dermoscopy, mycological testing, and nail clipping often contribute to a definitive diagnosis.^1^

Melanocytic pigmentation arises from the deposition of melanin in the nail plate, a condition referred to as true or Longitudinal Melanonychia (LM). LM occurs due to the activation or hyperplasia of melanocytes, primarily located in the nail matrix.^2^ This melanocyte hyperplasia can be benign (nevi and lentigo) or malignant (nail melanoma).

Tumoral lesions such as onychomatricoma, onychocytic matricoma, onicopapilloma, and Bowen's disease can also present with melanin pigment.^3^

In cases of LM, the differential diagnosis can be challenging and raises concern not only for general practitioners but also for dermatologists, given the possibility of early-stage nail melanoma.

Clinical and dermoscopic features, along with the progression of these lesions, offer valuable insights for making a more precise determination of cases warranting nail biopsy.

## Subungual hematoma

Subungual hematoma is one of the primary differential diagnoses for pigmented nail lesions. Although often underreported by patients, trauma stands as the leading cause, and its progression with nail plate growth serves as the key diagnostic evidence.

**Fig. 1:**
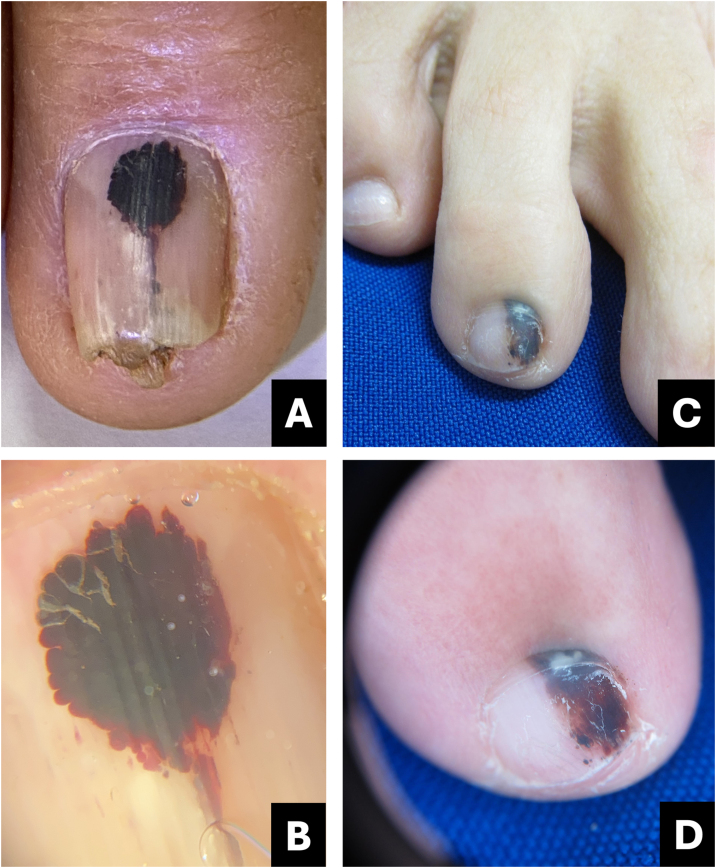
**Hematoma**. (A) Clinical: oval-shaped, blackish appearance; (B) Onychoscopy: purplish hue, globules and distal streaks; (C) Clinical: appearance of a darkened longitudinal streak; (D) Onychoscopy: purplish to bluish hue, sinuous borders, distal globules, pigment within the cuticle transparency simulating pseudo-Hutchinson and leukonychia at the site of trauma.

Clinically, it presents as an oval-shaped discoloration, ranging from reddish blue to bluish-black, with a more brownish hue in cases of older hematomas. It does not form a complete longitudinal band in the nail plate, distinguishing it from melanocytic-origin lesions (Fig. 1).^3^, ^4^

In onychoscopy, a homogeneous purplish color with a proximal globular pattern and distal streaking, which fades towards the periphery, becomes apparent. Leukonychia at the site of trauma is commonly observed (Fig. 1).^4^

Hematoma may be visible through the transparency of the cuticle, leading to confusion with the pseudo-Hutchinson sign. However, the hue and shape of the pigmentation assist in the differential diagnosis (Table 1).

**Table 1:** Comparative table between pigmented nail lesions.

	Clinical presentation	Onychoscopy	Tips
Subungual hematoma	Oval or incomplete streak	- Bluish-red or darkened blue;	Purplish, rounded, may spare the proximal region, progresses with nail growth.
		- Homogeneous, globular, or streaked pattern, fades at the periphery;	
		- Leukonychia (site of trauma).	
Onychomycosis	- Irregular linear;	- Multiple colors with yellowish-white streaks;	White or yellow streaks, inverted triangle, subungual hyperkeratosis, pigment may spare proximal region.
	- Multiple colors (from yellow to darkened);	- Irregular border in splinters;	
	- Irregular onycholysis;	- Northern Lights;	
	- Subungual hyperkeratosis.	- Subungual hyperkeratosis in ruins.	
*Pseudomonas* infection (onychobacteriosis)	- Oval or incomplete streak;	- Green to yellowish or blackish-blue;	Predominant color is green, associated with onycholysis ‒ usually located at the lateral edge of the nail.
	- Green to blackened.	- Areas of homogeneous coloring	
Melanocytic activation	- Regular linear.	Gray background associated with parallel, thin, and regular lines.	Gray background more visible to the naked eye than in onychoscopy. Free edge with pigment in the lower portion of the nail plate.
Benign melanocytic proliferation	- Regular linear with parallel streaks.	- Brown background color associated with regular brown lines in terms of parallelism, spacing, thickness, and color.	Brown background with regular streaks; Free edge with pigment in the upper portion of the nail plate.
Melanoma	- Irregular linear;	- Variation in color from brown to dark gray;	Brown background, gray to blackish with irregular streaks, without thickening or associated hyperkeratosis; Free edge with pigment in the upper portion of the nail plate.
	- Band >2/3 of the nail plate;	- Absence of parallelism in the lines;	
	- Hutchinson's sign;	- Incomplete lines;	
	- Nail dystrophy.	- Triangle sign.	
Bowen's disease	- Oblique linear;	- Longitudinal erythro/ leuko/ or melanonychia;	Irregular and non-parallel borders of the lesion; subungual hyperkeratosis.
	- Periungual verrucous lesion.	- Splinter hemorrhages;	
		- Localized subungual hyperkeratosis;	
		- Irregular and non-parallel borders;	
Onychomatricoma	- Well-defined linear;	- Distal parallel longitudinal leukonychia;	Localized thickening of the plate; swelling of the proximal fold, xanthonychia, and punctate holes in the distal plate.
	- Xanthonychia;	- Background erythema;	
	- Hypercurvature;	- Proximal splinter hemorrhages;	
	- Swelling of the proximal fold	- Thickening of the nail plate with distal punctate holes.	
		- Proximal hairpin vessels.	
Onychocytic matricoma	- Longitudinal pachymelanonychia.	- Well-defined longitudinal melanonychia;	Single lesion with localized thickening of the nail plate.
		- Subungual hyperkeratosis at the free edge.	
Onychopapilloma	- Regular linear;	- Longitudinal erythro/ leuko/or melanonychia;	Arises in the distal matrix andlocalized subungual hyperkeratosis.
	- From the lunula to the free edge.	- Pointed band from the distal matrix to the free edge;	
		- Splinter hemorrhages;	
		- Onycholysis with subungual hyperkeratotic mass.	

Diagnostic confusion between subungual hematoma and melanoma is common, especially when these lesions undergo digital dermoscopic analysis and are subsequently evaluated by software.

Subungual hematomas should be monitored until complete resolution. If bleeding persists in the same location a possible subungual tumor may be present. In such cases, imaging diagnosis, particularly through high-resolution/frequency Doppler ultrasound, aids in the diagnosis.

## Onychomycosis

Onychomycosis is the most prevalent nail disorder. The distal and lateral subtypes are the most common, leading to subungual hyperkeratosis, chromonychia, and onycholysis. Fungal invasion typically initiates in the hyponychium, an area of low adhesion of the nail plate, and progresses along the longitudinal ridges of the nail bed, resulting in a linear appearance of the fungal infection.^5^

In onychoscopy, the most frequent finding is an irregular border pattern with spicules at the proximal margin of the onycholytic area. Additionally, yellowish-white streaks in a “northern lights” pattern may also be observed.^5^, ^6^

**Fig. 2:**
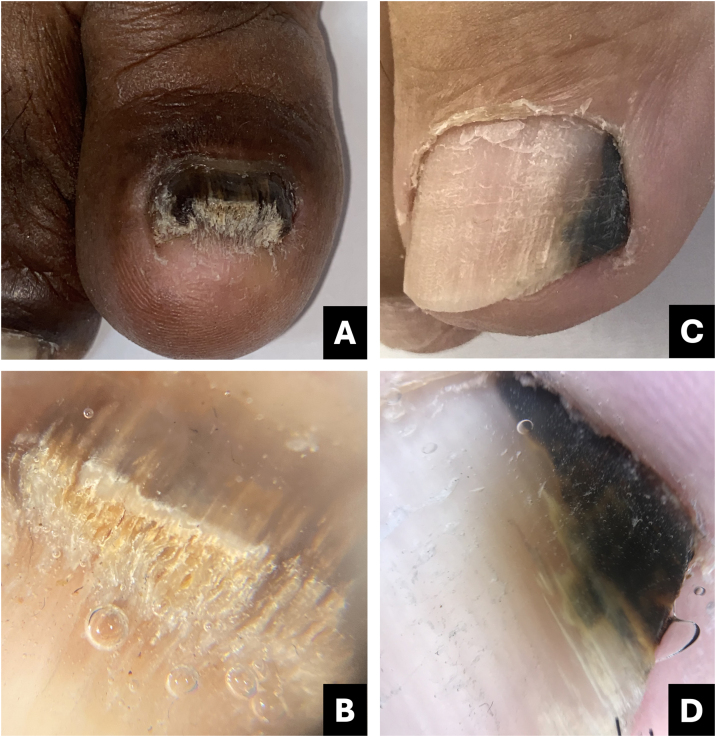
**Onychomycosis**. (A) Clinical: thickened and blackened nail; (B) Onychoscopy: subungual hyperkeratosis with ruin appearance, wide distal streaks, yellowish-white; (C) Clinical: irregular darkened band, wider at the distal portion (inverted triangle sign); (D) Onychoscopy: irregular black longitudinal streak, with wide yellowish-white distal streaks.

Onychoscopy of the free edge reveals thick subungual hyperkeratosis with ruin appearance, due to the accumulation of dermal debris reactive to the fungal invasion process (Fig. 2).^5^

Fungal melanonychia is caused by dematiaceous fungi, capable of producing melanin (*Scytalidium dimidiatum*, *Alternaria*, and *Exophiala*), as well as non-dematiaceous fungi (for example *Candida sp.* or *Trichophyton rubrum*). Dematiaceous fungi typically generate more diffuse pigmentation, while non-dematiaceous fungi induce melanocytic activation.^7^, ^8^

Some authors suggest that non-dematiaceous fungi induce inflammation and subsequent melanin production by the melanocytes of the nail matrix (hypermelanosis).^9^ However, there are studies supporting the idea that *Trichophyton rubrum* is capable of producing melanin or melanin-like compounds, both in vitro and in vivo.^10^

The clinical characteristic that distinguishes fungal melanonychia from nail melanoma is the presence of multiple colors, ranging from golden yellowish-brown to dark gray, predominantly clustered in the distal portion of the nail plate. Onychoscopic patterns suggestive of fungal pigmentation include a reverse triangular pattern (wider band distally than proximally, due to greater distal invasion of the fungus), subungual hyperkeratosis, scales on the nail surface, white or yellowish streaks, absence of visible melanin inclusions (granules < 0.1 mm), and homogeneous pigmentation (Fig. 2; Table 1).^7^, ^9^, ^11^

The diagnosis of onychomycosis necessitates mycological laboratory confirmation. Mycological examination (direct and culture) and/or nail clipping, followed by Periodic Acid-Schiff (PAS) staining or Polymerase Chain Reaction (PCR) is recommended for diagnostic confirmation.^12^

### *Pseudomonas aeruginosa* infection (onychobacteriosis)

*Pseudomonas aeruginosa* is a bacterium that can colonize the nail, inducing a greenish coloration often associated with onycholysis. The pigmentation arises from the production of the pigment pyocyanin by the bacteria. Clinically, the lesion ranges from light green to black, emphasizing the importance of distinguishing it from melanocytic pigmentation, particularly when affecting the lateral portion of the nail.^13^

The differential diagnosis encompasses subungual hematoma, malignant melanoma, and infections caused by other pathogens such as *Candida*, *Aspergillus*, as well as chemical exposure to solutions containing pyocyanin or pyoverdine.^14^

**Fig. 3:**
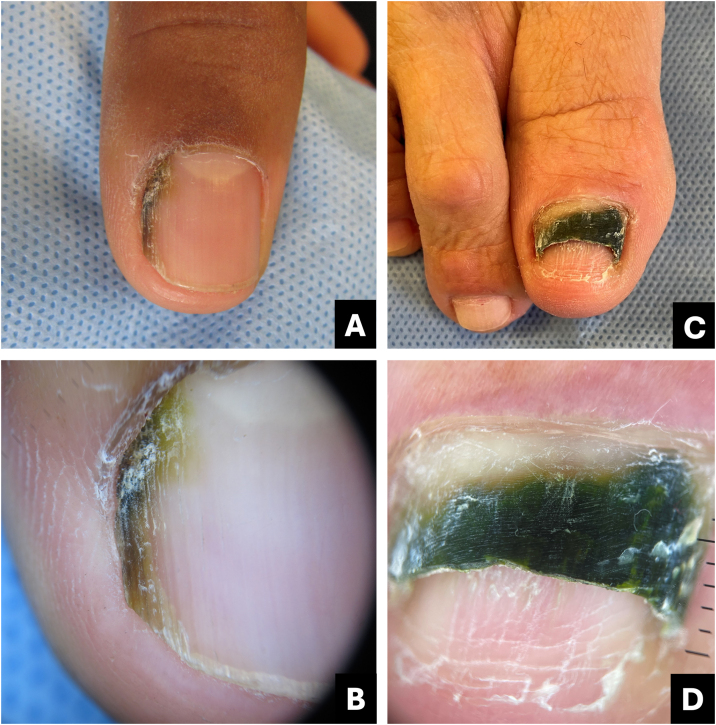
**Onychobacteriosis**. (A) Clinical: irregular brownish-black streak; (B) Onychoscopy: greenish-brownish-yellowish hue; (C) Clinical: dark greenish onycholysis; (D) Onychoscopy: dark greenish-yellowish color.

Onychoscopy aids in distinguishing exogenous pigmentation caused by *Pseudomonas*, which typically appears as bright green with a fading to pale green or yellow at the detached margin of the nail (Fig. 3; Table 1).

## True or Longitudinal Melanonychia (LM)

Melanocytes are sparsely distributed in the nail bed (approximately 50/mm^2^) and in the nail matrix (200/mm^2^), with higher activity observed in the distal matrix (L-DOPA positive).^2^, ^15^, ^16^, ^17^, ^18^, ^19^ These cells are typically quiescent in Caucasians and can become active due to various physiological and pathological conditions, or through drugs.^17^, ^20^

The number of melanocytes in the proximal matrix is equivalent to that in the distal matrix; however, they are incapable of producing pigment, unless there is proliferation and differentiation of benign melanocytes (nevi or lentigo) or malignant melanocytes (melanoma).

Melanonychia resulting from melanin deposition in the nail plate can occur due to melanocytic activation in the distal matrix (hypermelanosis) or melanocytic proliferation in the proximal matrix. Typically, it presents as a pigmented longitudinal band (LM), but it can on rare occasions affect the entire nail plate (total melanonychia).^17^, ^21^, ^22^

**Fig. 4:**
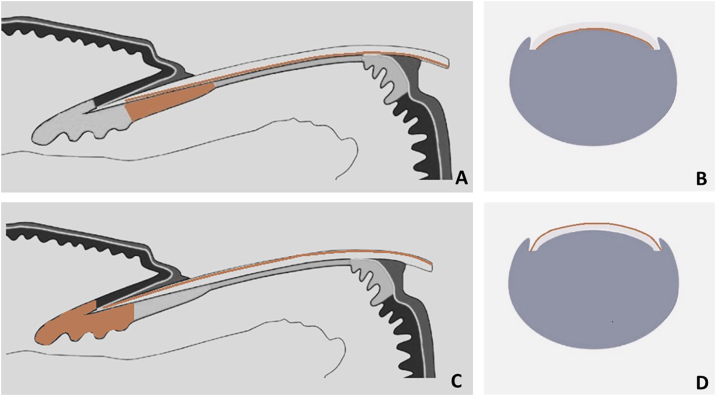
**Schematic drawing of matrix pigment in correlation to depth of nail plate pigmentation.** (A and B) Melanonychia with origin in distal nail matrix will be found in lower part of free nail edge. (C and D) Melanonychia with origin in proximal nail matrix will be found in upper part of free nail edge.

Surface and free-edge nail plate dermoscopy may be used to assist in the differential diagnosis of LM. The free edge examination aids in determining the position of the lesion in the matrix. Pigment located in the lower region of the nail plate indicates that the melanocytes producing melanin reside in the distal matrix, whereas pigment located in the superficial region indicates that the melanocytes producing melanin reside in the proximal matrix (Fig. 4). The entire thickness of the nail plate can be affected (Table 1).^1^, ^17^, ^21^

### Melanocytic activation (hypermelanosis)

This is the primary cause of LM in adults, particularly in dark-skinned individuals. It arises from heightened melanin production in the distal nail matrix, without melanocytic hyperplasia.^1^, ^17^, ^23^

**Fig. 5:**
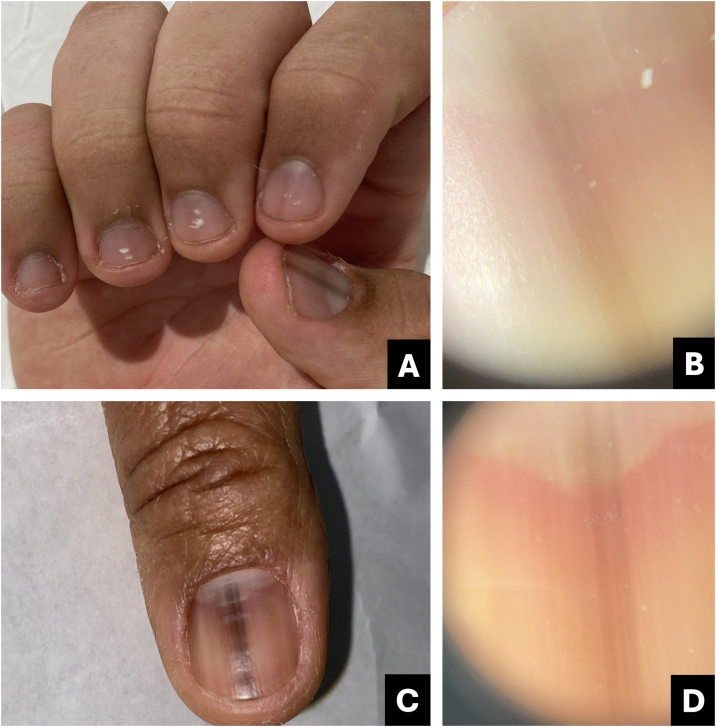
**Melanocytic activation.** (A) Clinical: multiple nails with gray to blackened streaks; (B) Onychoscopy: homogeneous gray streak lighter than is visible to the naked eye; (C) Clinical: Gray to darkened streak; (D) Onychoscopy: homogeneous gray streak lighter than is visible to the naked eye.

The number of nails affected is a relevant diagnostic clue. When multiple digits are involved, the primary consideration should be melanocytic activation (Fig. 5).^17^

The main described causes are physiological aspects (racial melanonychia and pregnancy), mechanical trauma, post-inflammatory melanonychia (post-inflammatory hyperpigmentation), local infections (fungal or bacterial), drug-induced melanonychia (hydroxyurea, tetracyclines, amlodipine, psoralens, zidovudine or some chemotherapeutic agents, mainly cyclophosphamide, doxorubicin, and capecitabine), systemic diseases (hemosiderosis, HIV, porphyria, alkaptonuria, Addison disease, and Cushing syndrome), specific syndromes (Laugier-Hunziker-Baran, and Peutz-Jeghers syndromes), iatrogenic (phototherapy), and associated with keratinocyte nail tumors (onychomatricoma, onychocytic matricoma, onychopapilloma, and Bowen disease).^17^, ^21^, ^24^, ^25^, ^26^

**Fig. 6:**
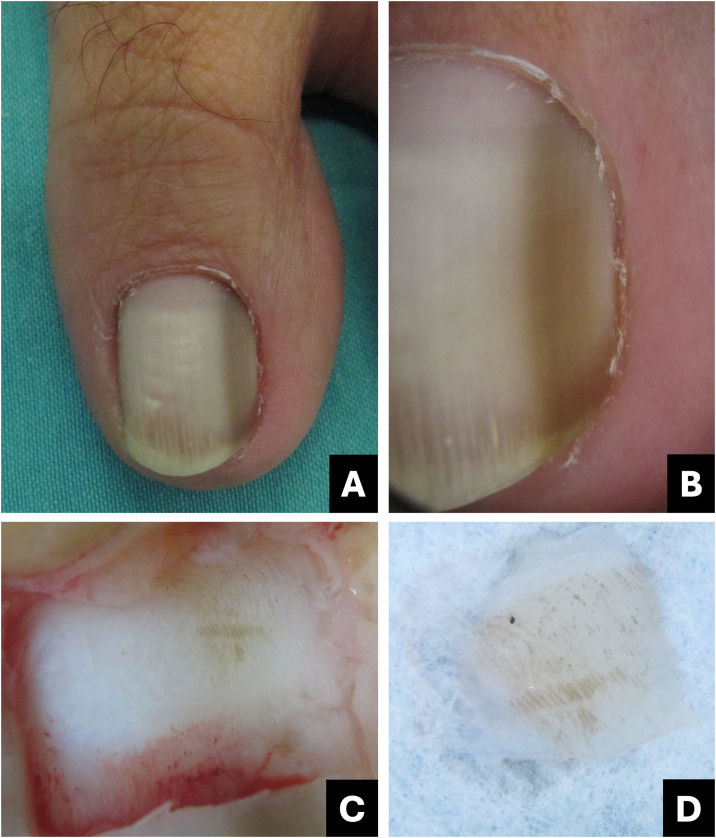
**Melanocytic activation.** (A) Clinical: brownish-gray longitudinal streak; (B) Onychoscopy: homogeneous brownish-gray pattern; (C) Intraoperative Onychoscopy: brownish-gray pigment with regular lines and some dots; (D) Ex vivo Onychoscopy: exhibits the in vivo findings.

Melanocytic activation is usually asymptomatic, and dermoscopy of the nail plate surface reveals, in the majority of cases, a gray background color associated with parallel, thin, and regular lines.^17^, ^27^, ^28^ However, in individuals with phototypes IV, V, and VI, it may manifest as a brown or black discoloration (Fig. 6).^29^ Free edge examination typically reveals pigment located in the lower region of the nail plate.^17^, ^27^, ^28^

Other distinctive features include the fact that the pigmentation color under dermoscopy is lighter when compared to the naked eye examination (Fig. 5, Fig. 6; Table 1), and the recurrence rate after biopsy or excision is high.

Intraoperative or *ex vivo* dermatoscopy reveals a regular gray pattern (Fig. 6).^30^ Some articles describe the possibility of visualizing greyish points corresponding to melanophages in histology.^31^

### Benign melanocytic proliferation

This group includes lentigo and melanocytic nevus and is characterized by melanocytic hyperplasia, which means there is a benign proliferation of melanocytes within the nail matrix. It presents as LM, with a color ranging from brown to black, and a width rarely exceeding 5 mm.^17^, ^21^, ^32^

**Fig. 7:**
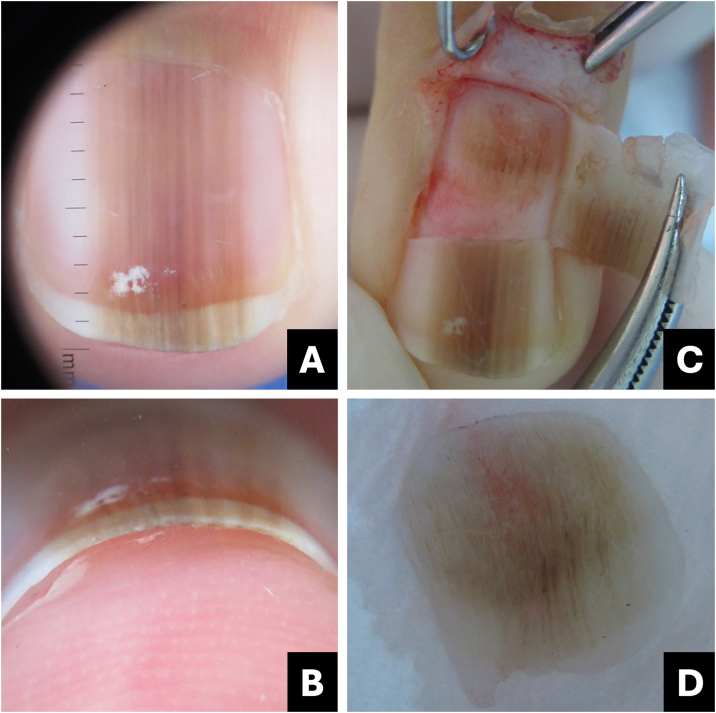
**Melanocytic nevus**. (A) Onychoscopy of the nail plate surface: light brown, dark, and gray longitudinal streaks, continuous individually and parallel to each other; (B) Onychoscopy of the free edge: pigment throughout the thickness of the nail plate; (C) Intraoperative Onychoscopy: regular lines with light brown pigment and some areas of thickening or blotches; (D) Ex vivo Onychoscopy: exhibits the in vivo findings.

**Fig. 8:**
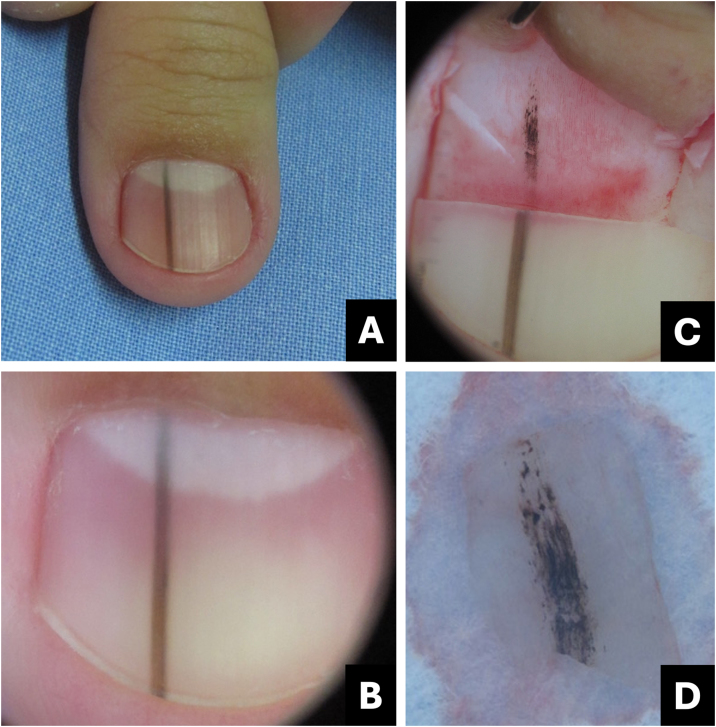
**Melanocytic nevus**. (A) Clinical: darkened longitudinal streak; (B) Onychoscopy: regular dark brown lines with tiny granules; (C) Intraoperative Onychoscopy: lines with darkened brown pigment and some globules or blotches; (D) Ex vivo Onychoscopy: exhibits the in vivo findings.

**Fig. 9:**
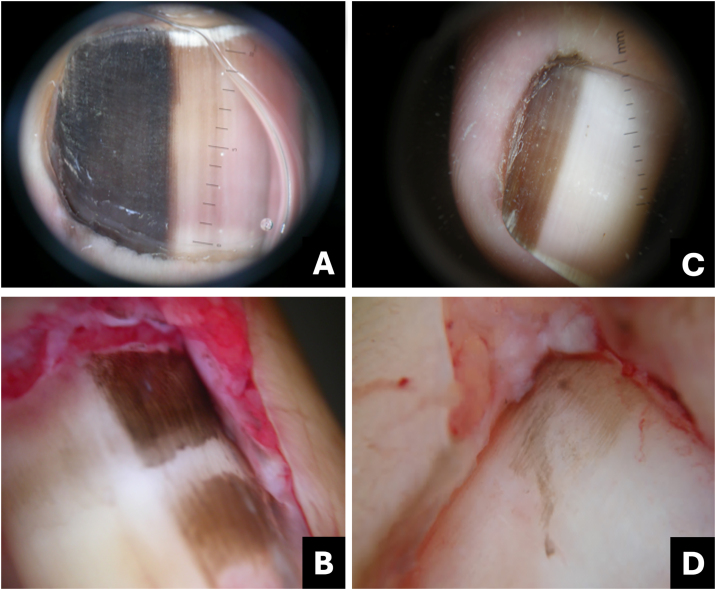
**Lentigo simplex**. (A) Onychoscopy: homogeneous darkened streak with regular brown streaks; (B) Intraoperative onychoscopy: homogeneous brown lines without blotches; (C) Onychoscopy: homogeneous, brown-blackened streak; (D) Intraoperative onychoscopy: homogeneous grayish-brown lines without blotches. (Courtesy of Dr. Sérgio Hirata).

A dermoscopy of the nail plate surface reveals a brown background color associated with regular brown lines in terms of parallelism, spacing, thickness, and color (Figs. 7‒9 ). Free edge examination typically reveals pigment located in the superficial region of the nail plate (Fig. 4; Table 1).^17^, ^21^, ^27^, ^28^

The differentiation between lentigo and melanocytic nevus is challenging and often necessitates nail matrix dermoscopy (intraoperative) and biopsy with subsequent histological examination. In terms of approach, it is not essential to differentiate between lentigo and nevus; however, distinguishing them from melanoma is always crucial.^33^

Intraoperative or *ex vivo* dermatoscopy reveals a regular brown pattern in the lentigo and a regular brown pattern with globules or blotches in the nevus (Figs. 7‒9).^30^

Histologically, the fundamental distinction between lentigo and nevus is that lentigo has a slight-to-moderate increase in typical melanocytes, without the presence of nests. In contrast, nevus exhibits the presence of nests of melanocytes and a variable number of individual melanocytes.^18^, ^34^, ^35^

### Melanoma (malignant melanocytic proliferation)

**Fig. 10:**
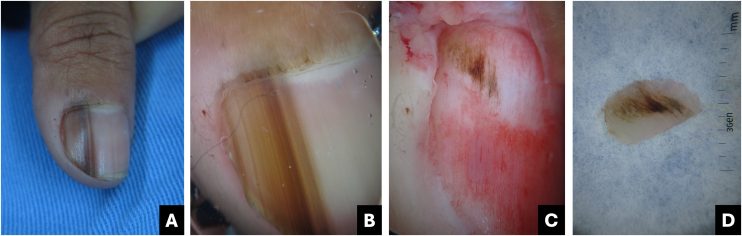
**Melanoma.** (A) Clinical: light brown to darkened longitudinal melanonychia; (B) Onychoscopy: light to dark brown streaks with apparent parallelism, pigment extends beyond the cuticle; (C) Intraoperative onychoscopy: irregular lines with light brown pigment, dark brown central amorphous area, and some asymmetric brown and darkened spots; (D) Ex vivo onychoscopy: exhibits the in vivo findings.

**Fig. 11:**
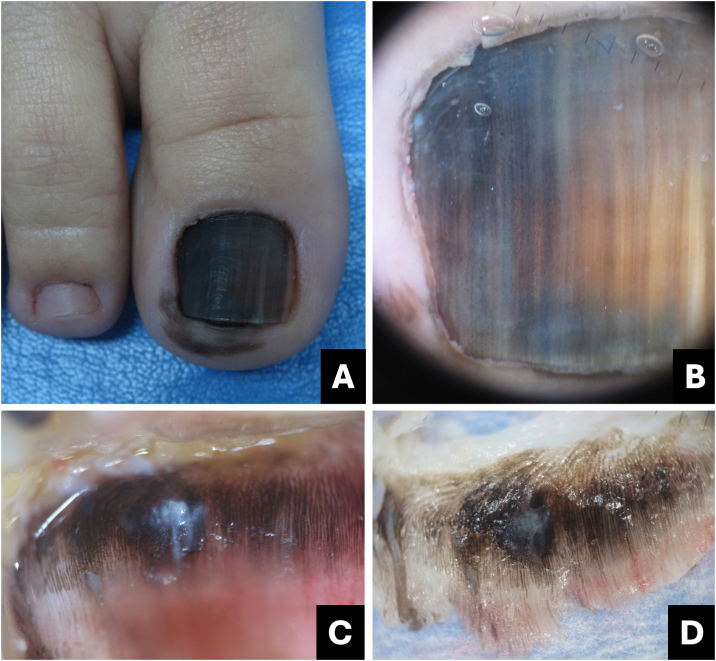
**Melanoma.** (A) Clinical: total blackened brown melanonychia with Hutchinson's sign (hyponychium); (B) Onychoscopy: dark brown to darkened streaks, some incomplete and non-parallel, with a gray-brown background; (C) Intraoperative onychoscopy: blackened irregular lines, with asymmetric distribution and a central grayish-white to blackened amorphous area. (D) Ex vivo Onychoscopy: exhibits the in vivo findings.

Melanoma is regarded as a subtype of acral lentiginous melanoma and typically originates within the nail matrix.^36^ In the early stages, nail melanoma may appear as LM (Fig. 10). The band may be wider proximally than distally (triangular).^37^ In its evolution, there may be periungual pigmentation, Hutchinson's sign (Fig. 11), not pathognomonic, but presumptive of melanoma, in addition to onychodystrophy which suggests a more advanced melanoma.^38^

Onychoscopy (nail plate surface) is characterized by a grayish-brown to the blackish background with irregular longitudinal lines in thickness, spacing, and color. Tiny, pigmented granules indicate melanocytic origin of lesions (Table 1).^3^ The width of the pigmented band varies, and it can progress to total melanonychia (Fig. 10, Fig. 11).^36^

A biopsy of the nail matrix may be indicated for adult patients with these characteristics, especially if growing LM is present in a single finger.^9^

Intraoperative or *ex vivo* dermoscopy reveals lines with irregularity in color and thickness, as well as irregular globules and blotches (Fig. 10, Fig. 11).^30^

The histological findings, in summary, would be an increased number of melanocytes with atypia (both as individual cells and in nests), pagetoid infiltration, and neoplastic infiltration of the dermis, which distinguishes the invasive form from the *in situ* form.^18^, ^34^, ^35^

Recent articles report the possibility of a preliminary assessment through nail clipping and histopathological examination, by observing isolated atypical melanocytes in the nail plate.^39^

## Bowen's disease

Bowen’s disease is the most common malignant tumor of the nail apparatus. It corresponds to *in situ* squamous cell carcinoma and is usually associated with high-risk *Human Papillomavirus* (HPV), including types 16, 18, 35, and 56. Men aged 35 to 60 are predominantly affected. It typically involves only one finger, with the thumb being the most common localization (44%).^40^

**Fig. 12:**
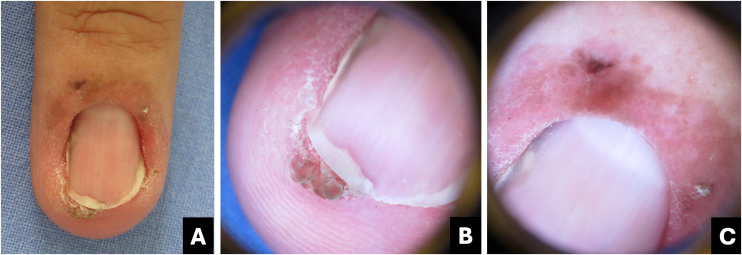
**Bowen's disease.** (A) Clinical: erythematous patch with brown pigment in the proximal fold, lateral subungual hyperkeratosis associated with distal onycholysis and streaked melanonychia; (B) Onychoscopy of the free edge: localized subungual hyperkeratosis associated with onycholysis and oblique grayish-brown pigment streak; (C) Onychoscopy (proximal fold): linear brown dots associated with dotted vessels.

The nail bed is most affected, resulting in lateral onycholysis with a verrucous lesion and LM. Nail bed erosion, nodulation, and in rare cases, longitudinal erythronychia may be present. Involvement of the nail fold results in an irregular erythematous patch or plaque (Fig. 12). Melanonychia is more frequent in individuals with dark skin and in cases associated with HPV-56.^41^, ^42^

The onychoscopy pattern of pigmented Bowen's disease is characterized by linear brownish dots or a clustered glomerular vascular pattern. Other findings include localized subungual hyperkeratosis, irregular non-parallel erythro/leukonychia or LM, and splinter hemorrhages (Fig. 12).^4^, ^43^

Melanonychia forms an oblique line from proximal to distal, associated with hyperkeratosis, which aids in the differential diagnosis between pigmented Bowen's disease and nevi and/or nail melanoma (Table 1) (Fig. 12).^44^

The biopsy with histopathological examination, after avulsion of the nail plate and exposure of the tumor, is mandatory for diagnosis.^42^

The loss of normal epidermal stratification, dyskeratosis, clustered large cells with hyperchromatic nuclei, and atypical mitoses are characteristic histopathological findings. When associated with HPV, perinuclear vacuolization is typically observed.^38^ *In situ* hybridization is recommended for HPV identification.

## Onychomatricoma

Onychomatricoma is a benign fibroepithelial tumor of the nail matrix. It is evenly distributed between sexes, with an average age of onset at 48 years. The fingers are most commonly affected. It is characterized by an overproduction of nail plate localized within the tumor's topography, resulting in thickening, xanthonychia, and transverse and longitudinal hypercurvature of the nail plate.^45^

**Fig. 13:**
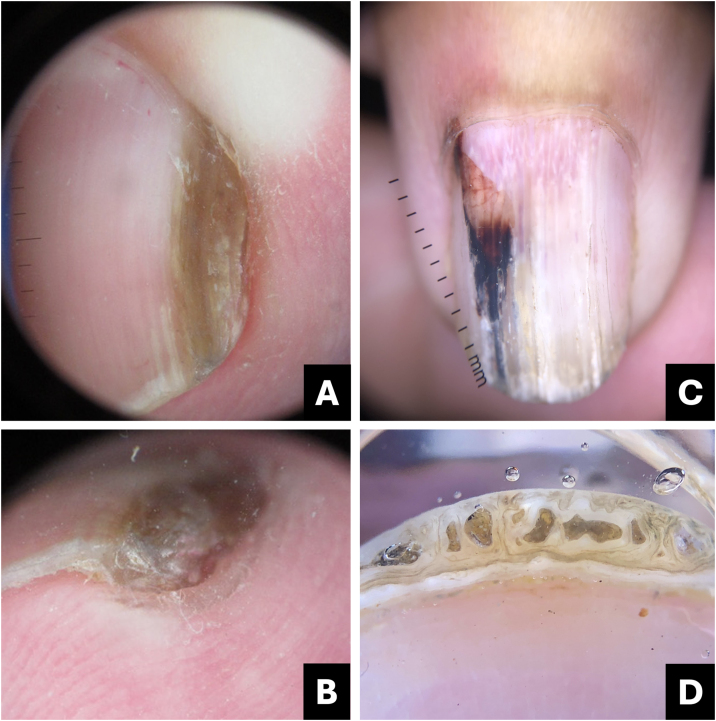
**Onychomatricoma.** (A) Onychoscopy (nail plate surface): homogeneous brownish longitudinal streak; (B) Onychoscopy (free edge): thickening of the nail plate; (C) Onychoscopy (nail plate surface): white longitudinal streaks, proximal dilated vessels, irregular red to darkened pigment; (D) Free edge: thickening of the plate with holes.

A swelling of the proximal fold can be observed, beneath which the tumor is located. Splinter hemorrhages in the proximal region of the nail plate occur due to the projection of connective tissue fingers with blood capillaries from the tumor, which also form longitudinal channels within the nail plate (Fig. 13). The thickened free edge has small openings that may still contain tumor projections and patent capillaries, potentially causing bleeding when cutting the nail.^46^

Onychoscopy reveals a parallel longitudinal leukonychia (due to air in the channels of the nail plate), splinter hemorrhages (more frequently proximal), background erythema, staple-shaped vessels, and thickening of the nail plate with distal perforations (Fig. 13; Table 1).^43^

There may be dark pigmentation; in these cases, melanonychia is associated with localized thickening of the nail plate and perforations (free edge), aiding in differentiation from nail melanoma (Fig. 13; Table 1).^25^, ^47^ Nail clipping, ultrasonography, and magnetic resonance imaging assist in the diagnosis.^48^

Histologically, the distal zone is characterized by several finger-like papillary projections, covered by matrix epithelium. On the other hand, the proximal zone is lined with matrix epithelium featuring papillomatous characteristics, with deep invaginations vertically oriented in the stroma. The stroma is moderately to highly cellular, composed of fibroblastic cells with random orientation and an increased number of mast cells.^18^

## Onychocytic matricoma

Onychocytic matricoma was described in 2012 by Perrin et al. upon observing 5 cases of longitudinal pachymelanonychia, in which the histological findings were of “acanthoma of the nail matrix producing onychocytes”.^49^ All cases described thus far have occurred on the fingers, as a single lesion, characterized by localized thickening of the nail plate (pachyonychia), longitudinally brownish melanonychia (variable pigmentation from mild to intense), and subungual keratosis at the free edge.^38^, ^49^, ^50^, ^51^, ^52^ Only one case was described as a hypopigmented variant, in which xanthonychia was observed instead of melanonychia.^53^

**Fig. 14:**
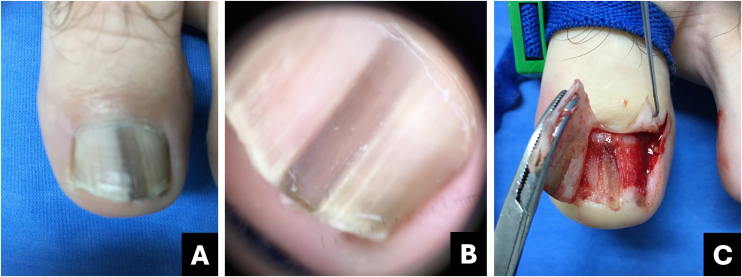
**Onychocytic matricoma:** (A) Clinical: homogeneous gray-blackened longitudinal streak; (B) Onychoscopy: homogeneous gray-blackened longitudinal streak with subungual hyperkeratosis; (C) Intraoperative: thick, pigmented linear tumor from the matrix to the hyponychium.

A well-defined longitudinal melanonychia and subungual keratosis at the free edge/hyponychium are better visualized with dermoscopy (Fig. 14; Table 1).^50^

Histologically, it consists of a proliferation of the basal layer of matrix cells, similar to seborrheic keratosis. It is predominantly composed of basophilic cuboidal cells. Eosinophilic nail pearls (keratinous inclusions) surrounded by a pre-keratogenic zone and a keratogenic zone are observed.^38^ In the papillomatous type, numerous small epithelial projections covered by a thin keratogenic zone are seen.^52^

## Onychopapilloma

This is a benign neoplasm of the nail bed and the distal portion of the matrix. Clinically, it can present as longitudinal erythronychia, melanonychia, or leukonychia, measuring 0.3 to 1.5 mm, extending linearly from the distal matrix (lunula) to the free edge.^54^

**Fig. 15:**
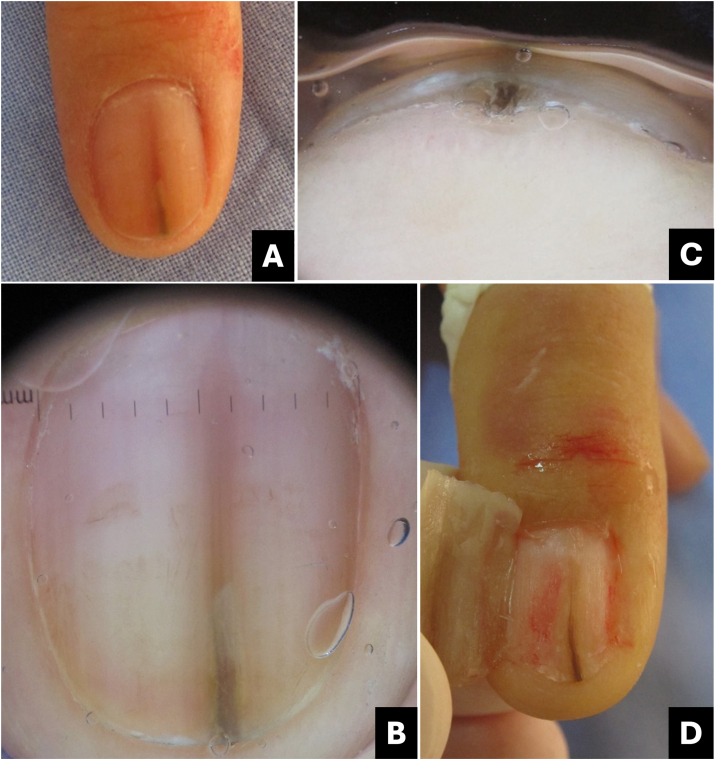
**Onychopapilloma.** (A) Clinical: longitudinal erythro/melanonychia; (B) Onychoscopy (nail plate surface): pointed erythro/melanonychia at the lunula; (C) Onychoscopy (free edge): localized subungual hyperkeratosis. (D) Intraoperative: pigmented filiform tumor from the distal matrix to the hyponychium.

Generally, distal subungual hyperkeratosis is observed beneath the band projection (Fig. 15). As the tumor originates in the distal matrix, which is involved in nail formation, the plate is slightly thinner above the tumor, which can lead to distal fissuring.^38^

Onychoscopy of the free edge shows a subungual keratotic mass. When examining the nail plate surface, it emphasizes the pointed band extending from the distal matrix to the free edge, accompanied by splinter hemorrhages and distal onycholysis (Fig. 15; Table 1).^46^

Surgical treatment may be considered if there is discomfort due to the distal plate fissure or to rule out other differential diagnoses, such as squamous cell carcinoma or nail melanoma.

In histological examination, the matrix area of the tumor displays an endo-exophytic acanthoma, characterized by deep clefts and protrusion into the adjacent nail. The tumor produces intensely eosinophilic keratin, in contrast to the healthy nail. In the nail bed, the tumor flattens, and the dermis often exhibits numerous small inclusions, some solid and others corresponding to small onycholytic cysts.^38^

## Conclusion

Pigmented nail lesions remain a challenge for general practitioners and dermatologists.

Knowledge and observation of clinical and onychoscopic characteristics, as well as the progression of the lesion, in conjunction with other supplementary examinations, assist in differentiating nail melanoma from other pigmented nail lesions. However, in cases where doubt persists, histopathological examination remains the gold standard.

## Financial support

None declared.

## Authors’ contributions

Laura Bertanha: Participated in generating and analyzing the data; wrote the majority of the original draft of the paper; reviewed the pertinent raw data on which the results and conclusions of this study are based and approved the final version of this paper.

Leandro Fonseca Noriega: Participated in generating and analyzing the data; wrote the majority of the original draft of the paper; approved the final version of this paper.

Nilton Gióia Di Chiacchio: Participated in generating data; writing the paper and approving the final version of this paper.

Adriana Matter: Participated in generating and analyzing the data; wrote part of the original draft of the paper; approved the final version of this paper.

Nilton Di Chiacchio: Participated in generating data; writing the paper; reviewing the pertinent raw data on which the results and conclusions of this study are based and approved the final version of this paper.

## Conflicts of interest

None declared.
